# Structural and Optical Characteristics of PVA:C-Dot Composites: Tuning the Absorption of Ultra Violet (UV) Region

**DOI:** 10.3390/nano9020216

**Published:** 2019-02-06

**Authors:** Shujahadeen B. Aziz, Aso Q. Hassan, Sewara J. Mohammed, Wrya O. Karim, M. F. Z. Kadir, H. A. Tajuddin, N. N. M. Y. Chan

**Affiliations:** 1 Advanced Polymeric Materials Research Laboratory, Department of Physics, College of Science, University of Sulaimani, Qlyasan Street, Sulaimani 46001, Kurdistan Regional Government, Iraq; 2 Komar Research Center (KRC), Komar University of Science and Technology, Sulaimani 46001, Kurdistan Regional Government, Iraq; 3 Department of Chemistry, College of Science, University of Sulaimani, Qlyasan Street, Sulaimani 46001, Kurdistan Regional Government, Iraq; aso.hassan@univsul.edu.iq (A.Q.H.); sewara.mohammed@univsul.edu.iq (S.J.M.); wrya.karim@univsul.edu.iq (W.O.K.); 4 Centre for Foundation Studies in Science, University of Malaya, Kuala Lumpur 50603, Malaysia; mfzkadir@um.edu.my; 5 Department of Chemistry, College of Science, University of Malaya, Kuala Lumpur 50603, Malaysia; hairul@um.edu.my (H.A.T.); nadianabihahchan@gmail.com (N.N.M.Y.C.)

**Keywords:** carbon nanodots, hybrid polymer composites, FTIR study, XRD study, optical properties

## Abstract

In this work the influence of carbon nano-dots (CNDs) on absorption of ultra violet (UV) spectra in hybrid PVA based composites was studied. The FTIR results reveal the complex formation between PVA and CNDs. The shifting was observed in XRD spectrum of PVA:CNDs composites compared to pure PVA. The Debye-Scherrer formula was used to calculate the crystallite size of CNDs and crystalline phases of pure PVA and PVA:CNDs composites. The FESEM images emphasized the presence and dispersion of C-dots on the surface of the composite samples. From the images, a strong and clear absorption was noticed in the spectra. The strong absorption that appeared peaks at 280 nm and 430 nm can be ascribed to the n-π* and π-π* transitions, respectively. The absorption edge shifted to lower photon energy sides with increasing CNDs. The luminescence behavior of PVA:CNDs composite was confirmed using digital and photo luminescence (PL) measurements. The optical dielectric constant which is related to the density of states was studied and the optical band gap was characterized accurately using optical dielectric loss parameter. The Taucs model was used to determine the type of electronic transition in the samples.

## 1. Introduction

Since the invention of carbon nano-tubes (CNTs), carbon-based nano-materials have been widely investigated. Carbon quantum dots (CQDs) currently represent the newest class of carbon-based materials as a potential alternative to CNTs for sustainable applications [1]. Carbon nano-dots (CNDs) as a new carbon nano-material with discrete, quasi-spherical carbon nano-particles and ultrafine size of almost 10 nm can be used as a building block for fluorescence systems [2]. Several advantageous characteristics of C-dots, such as an abundance of carbon sources, low cost, biodegradability and brilliant fluorescence behavior, make these new materials widely applicable. Moreover, chemical stability in the colloidal solution state, inertness and relatively high resistivity to photo-bleaching also make C-dots superior over traditional fluorescent organic dyes and semiconductor quantum dots [3]. Recently, has obviously been shown that CNDs have diversity in applications, for instance for biomedical imaging, catalysis, bio-imaging, drug delivery, energy, photovoltaic devices and optoelectronic purposes [2,3]. Because of the abundance of oxygen/hydrogen-containing species such as –OH and –COOH on the surfaces of CNDs, they are chosen as fillers to enhance the hydrogen bonding [4]. Lately, carbon nano-dots (CNDs) have emerged as a new family of light-harvesting materials with remarkable advantages including strong and broad optical absorption, high chemical stability, excellent electron- and hole-transfer capability, and low toxicity [5]. The incorporation of CDs within polymer matrices is under intense study and thus can be utilized in many photonic and optoelectronic applications and integrated in real devices [6]. Organic–inorganic composites are designed for new eras of optical, nonlinear optical, electronic devices and biological labels [7]. As far as we know, polymer nano-composites have attracted the attention of many research groups because of their unique physicochemical properties and wide applications. Moreover, the incorporation of C-dots into solid polymer matrices prevents nano-particles coagulation; as a consequence, higher stability is noticed relative to the colloidal solution counterparts [8]. It is well-known that before applying the organic composites into the devices, their optical properties and morphological profiles have to be characterized. This is due to the fact that any tiny alteration may cause fluctuation in the performance [9]. Herein, poly (vinyl alcohol) (PVA) is considered an attracting artificial polymer that is benign and water soluble, in addition to a relatively high dielectric constant and an excellent film forming capability. All these valuable properties of this polymer can be ascribed to this backbone structure that enables it to form hydrogen bond; as a result, hydrophilic nature dominates and cross linking ability increases with the doping materials [10,11]. In the past decade, immense focus has been devoted to the composites with high transparency and luminescence behavior which have various applications in light emitting devices. Specifically, CD semiconductors are superior to the others in terms of light stability and low toxicity [12]. However, the luminescence-quenching process induced by the particle aggregation limits the application of CDs concerning color tunability and white light fabrication in solid-state illumination systems. The aggregation of CD particles can effectively be avoided via combination with polar polymers [13]. In the current work, a polar, thermo-stable, chemical resistive, easily processible and transparent PVA was used as a hosting polymer in the fabrication of the composite [8]. Recently, extensive research interest has been focused on the improving understanding of the nature of charge transport from the valence to conduction band. This involves attempts to synthesize polymer composites with different ratios between CNDs and the PVA hosting polymer.

## 2. Synthesis of CDs and Preparation of Polymer Composites

PVA used in this study was supplied by Sigma-Aldrich (Kuala Lumpur, Malaysia). PVA: CNDs polymer nanocomposite films were prepared by the well-known solution casting technique. Hydrothermal treatment of glucose resulted in the formation of the yellow carbon nano-dots (CNDs), as follows: 1 g of glucose was dissolved in 5 mL of concentrated phosphoric acid and the resulting solution was colorless. The solution was then heated in a water bath at (80–90) °C for (20–30) minutes until a dark brown solution was obtained. The solution was cooled down to the room temperature and the pH was adjusted between 3 and 4 using dilute NaOH, afterwards, it was left overnight. The purification of CNDs was conducted using chloroform and then evaporation of the chloroform was performed. The mass of the synthesized CNDs (5 mg) was obtained by subtracting the mass of the beaker from the one of the beakers plus the CNDs. A homogeneous solution of CNDs was obtained by adding 45 mL of distilled water to the CNDs with continuous stirring.

In the preparation of PVA solution, 1 g of PVA was dissolved in 50 mL of distilled water. Afterwards, it was left under continuous stirring at room temperature for 24 hrs until the whole polymer was completely dissolved. As a result, a clear and viscous solution was gained. The final step is preparation of the polymer nano-composite by adding a different portion of CD into a separate container containing PVA solution under a continuous stirring condition. All sample solutions are labeled as CND0, CND1 and CND2 correspond to incorporated PVA solution with 0 mL, 15 mL and 30 mL of 5 mg of dissolved CNDs, respectively. These solutions are further stirred until a homogenous state was achieved. The samples were casted in Petri dishes, and then left for drying at room temperature to allow the film to be formed. The film thickness was controlled in the range of 120–121 μm using constant amount of PVA. Further drying was obtained by transferring the sample solutions into desiccators in an attempt to gain solvent free-films.

## 3. Characterization Techniques

X-ray diffraction (XRD) data were collected at room temperature using a diffractometer (Bruker AXS, Billerica, MA, USA) operating at a voltage of 40 kV and a current of 40 mA. The samples were scanned with a monochromatic X-radiation beam of wavelength λ = 1.5406 Å and the glancing angles were in the range of 5° ≤ 2θ ≤ 80° with a step size of 0.1°. UV-vis absorption spectra were measured on a Jasco V-570 UV-Vis-NIR spectrophotometer (Jasco SLM-468, Tokyo, Japan) in the absorbance mode. The formation of CNDs-PVA complexes was investigated by Fourier-transform infrared (FTIR) spectroscopy. FTIR spectra were recorded on a Nicolet iS10 FTIR spectrophotometer (Thermo Fischer Scientific, Waltham, MA, USA) in the wave number range of 4000–400 cm^−1^ with a resolution of 2 cm^−1^. The ATR method was used to measure the FTIR spectrum of the films. The surface morphologies of the PVA:CND composites were examined using Hitachi SU8220 field emission scanning electron microscopy (FESEM) (Europark Fichtenhain A12, 47807 Krefeld, Germany).

## 4. Results and Discussion

### 4.1. FTIR Study

**Figure 1:**
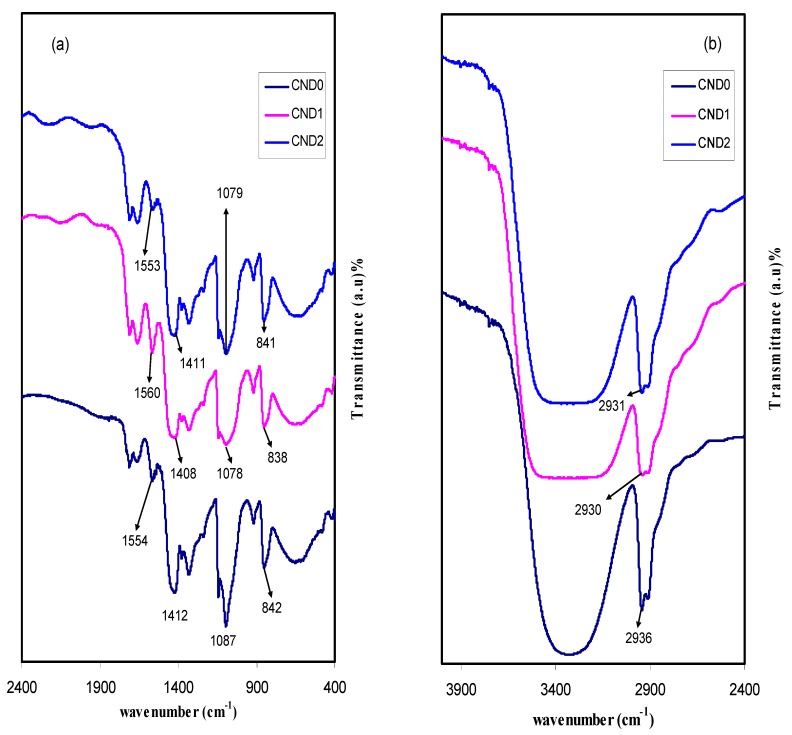
FTIR spectra of all samples in the region of (**a**) 400 cm^−1^ to 2400 cm^−1^, and (**b**) 2400 cm^−1^ to 4000 cm^−1^. Clear shifting, broadening and change in intensity in the FTIR bands can be observed.

FTIR analyses were used to investigate the complex formation in the samples. The FTIR spectra of pure PVA and all the prepared PVA:CND composites are depicted in Figure 1. From the spectrum, the stretching vibration of the hydroxyl groups (O–H) of the pure PVA [14] peaked at 3322 cm^−1^ which increased in intensity and broadness as a result of increasing carbon nano-dots, in CND1 and CND2 doped samples. The wide dispersion of hydrogen bond donor groups, such as –OH and –COOH over the CND surfaces resulted in broadness of the FTIR at 3322 cm^−1^. This is well-defined that the hydrogen bonding changes both the position and shape of the IR absorption band [4]. Meanwhile, a complex formation between the PVA and CNDs is evidenced from this peak broadening and intensity attenuating. Moreover, the shift of C–H stretching of CH_2_ group of pure PVA from 2936 cm^−1^ [15], to 2930 and 2931 cm^−1^ was observed in CND1 and CND2, respectively. In addition, there is another shift in C–H bending peak position of pure PVA from 1412 cm^−1^ to 1408 cm^−1^ and 1411 cm^−1^ in CND1 and CND2, respectively. The C–O bending and stretching of the acetyl group on the polymer backbone [16,17] appeared at 1087 cm^−1^ in PVA which also shifted to 1087 cm^−1^ and 1079 cm^−1^ in the respective samples. A vibration peak located between 842 cm^−1^ and 841 cm^−1^ can be ascribed to either C–H rocking mode or C–C stretching [16,18]. Two weak absorption peaks are seen at 1740 cm^−1^ and1370 cm^−1^, indicating vibrational stretching of C=O and CH_3_– in acetate moiety which in turn ascribed to incomplete alcoholysis [19]. A relatively sharp peak can clearly been seen at 1561 cm^−1^, indicating skeletal N–H bending mode [20]. All these changes in the peak positions in the IR spectrum indicate sufficient cross-linking between PVA and CND nano-particles.

### 4.2. XRD and Morphology Study

**Figure 2:**
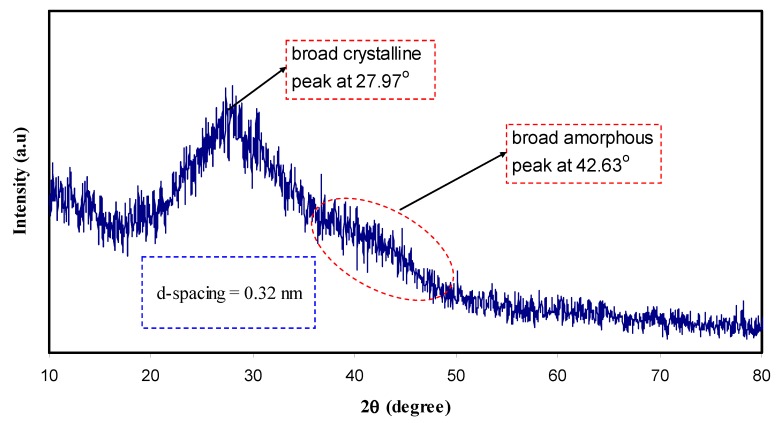
XRD pattern of CN-dots at ambient temperature. Crystalline and amorphous peaks can be seen in the XRD spectrum.

**Figure 3:**
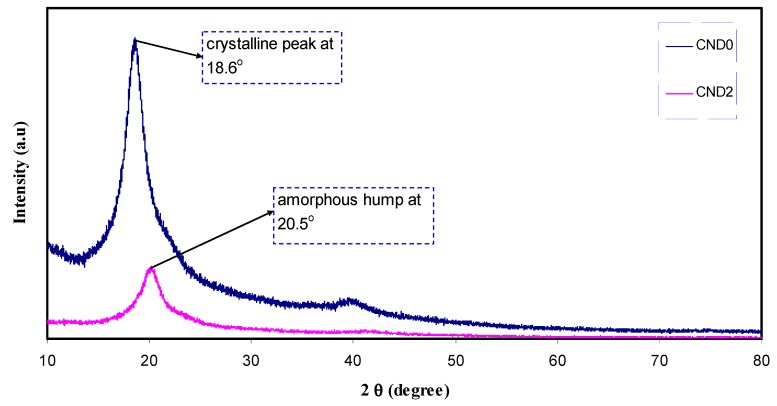
XRD pattern of pure PVA and PVA:CN-Dot composite films. It is interesting to note that the main peak of PVA is more broadened and it intensity decreased after incorporation of CN-dots.

Figure 2 shows the XRD spectrum of CND particles. It is clear that CDs exhibits a broad crystalline peak at about 2θ = 27.97° and a broad amorphous peak at 42.63°. Previous studies attributed the former peak to highly disordered carbon atoms [21]. Figure 3 represents the XRD pattern of pure PVA and PVA doped with CNDs particles. As can be seen, a broad peak at around 2θ = 20° in pure PVA corresponds to the semi-crystalline nature of the polymer [22]. The XRD pattern (Figure 2) obtained for the C-dots in this work is completely different from that of former work which is relatively broader [23]. For the C-dots obtained, the d-spacing value (0.32) is smaller than that reported (0.34) in the literature [2,23]. It has been proved that the broad peaks in the XRD pattern suggest the nano-scale nature of the prepared particles [24,25,26]. This can be understood mathematically from the well-known Debye-Scherrer formula:

*L = Kλ/β cosθ* (1)

This means the broader the diffraction peaks are the larger full width at half maximum (*β*) which in turn led to a smaller crystallite size (*L*) [27,28]. From Equation (1) the crystallite size was calculated manually using *λ* = 1.5406 Å, *K* = 0.9 and the full width at half maximum (*β*) from the main peak of the XRD pattern at specified 2θ can be estimated. The crystalline size estimated from Equation (1) for the largest peak in the XRD pattern (2θ = 27.97°) of Figure 2 is 1 nm for carbon nano dote (CND) particles. Therefore, the small size of the C-dots particles is proved from the broad XRD peak. The characteristic feature of C-dots is carbogenic core consisting of both amorphous and crystalline structural parts which enrich in surface functional groups. It is worth-mentioning that in C-dots, amorphous part dominates [2]. 

The XRD patterns for pure PVA and the PVA doped (CND2) samples (see Figure 3) are evidences for the formation of complexation between PVA and CNDs particles. The relatively large peak centered at 18.6° is shifted to 20.5° for PVA doped. Another two broad peaks appeared at 2θ = 23.4° and 41.18°. From the literature, one can expect that 2θ = 20°, 23.43° and 41.15° belong to (101), (200) and (111) crystalline phases of PVA [19] and these shifts also result from complexation between functional groups of PVA and surface groups of CNDs particles. This study showed that as the concentration of CNDs increased, the intensity decreased and the peaks underwent broadening. These results were caused by the disruption of hydrogen bonding between the surface groups of CNDs and the hydroxyl group in PVA polymer, resulting in it dominating the amorphous part of the composites [14,29]. The calculated crystalline size from equation (1) for peaks 2θ = 18.6° and 20.5° are 6.5 and 4.6 nm in pure PVA (CDN0) and PVA doped one (CND2), respectively. Thus the crystallite size of regular phases or chains of PVA in crystalline regions was reduced upon addition of CNDs particles to PVA. The strong evidences of amorphous domination PVA doped one (CND2) are lowering in intensity and broadening the peaks. The non-existence of peaks for CNDs in PVA doped sample indicates the dissolution of the whole CNDs in the polymer matrix.

**Figure 4:**
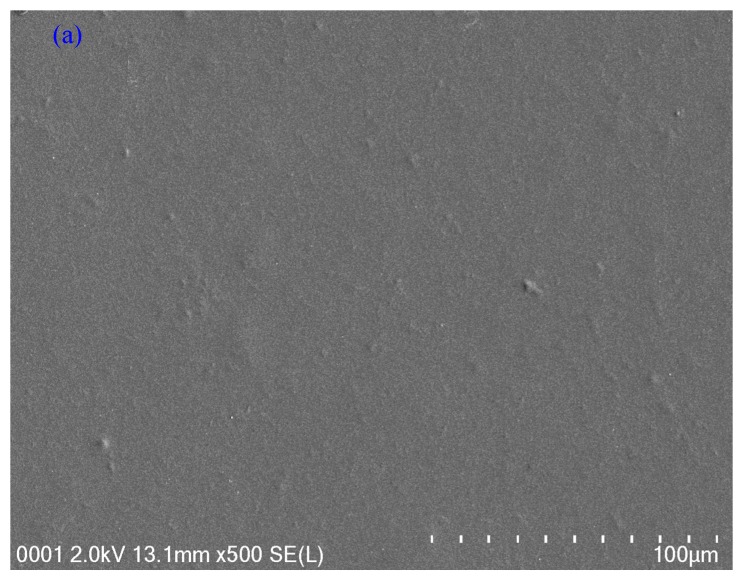

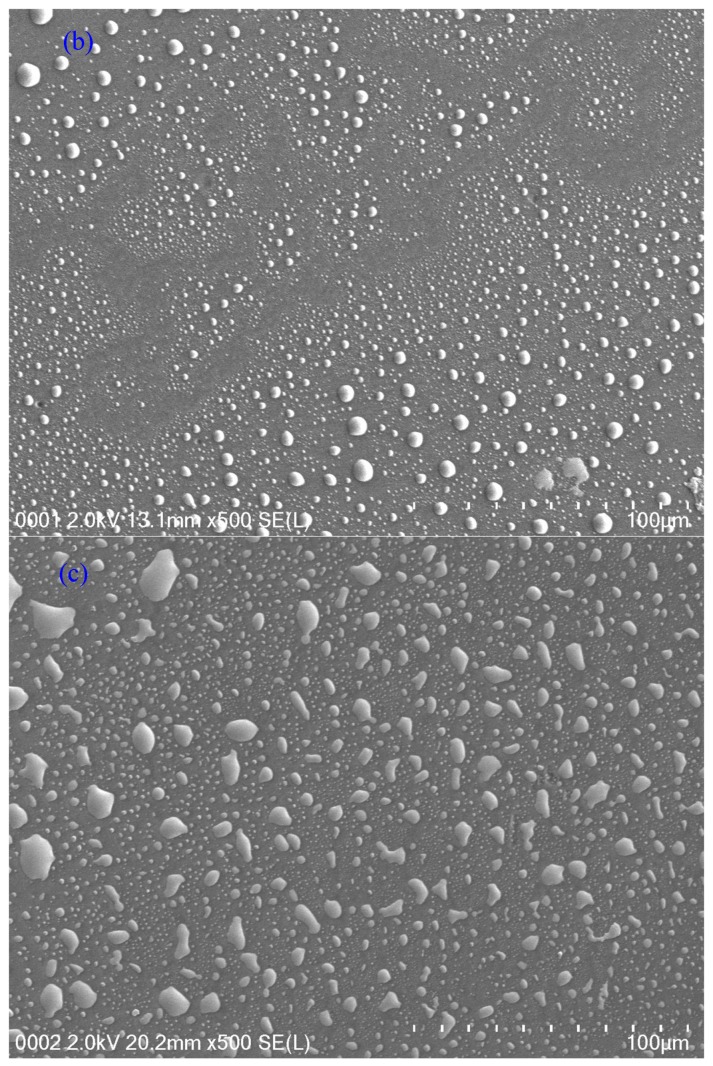
SEM images for (**a**) CND0, (**b**) CND1 and (**c**) CND2 samples.

To know the extent of compatibility between the polymer and fillers and also the leakage of the nano-particles to the polymer surface, a commonly utilized technique is field emission scanning electron microscopy (FESEM) [30,31,32]. The surface morphology was studied using field emission scanning electron microscopy (FESEM). The surface images have shown the formation and distribution of PVA: CND polymer composite. Figure 4a–c show the acquired surface images of both pure PVA and PVA: CND composites, respectively. From the images, one can clearly see the presence and distribution of C-dots on the composite surfaces. Figure 4c exhibits the FESEM image of CND2 sample which incorporated with 30 mL of suspended CND filler. From the image, it is seen that large size CND particles formed on the surface. The FTIR spectra have confirmed the existence of various functional groups, such as –OH and –COOH on the surfaces of CNDs which are incorporated as fillers to enhance hydrogen bonding ability [4]. Another observation that has to be taken into consideration is high density of –OH distribution homogeneously in the polymer [33]. This homogenous distribution infers adequate interfacial interaction between C-dots and polymer matrix as a result of hydrogen bond formation. Therefore, the larger size particles are produced at high concentration. The atomic force microscopy (AFM) has shown the roughness of the surface of PVA:CQD composite at high content of C-dot filler [4].

### 4.3. Absorption Study

**Figure 5:**
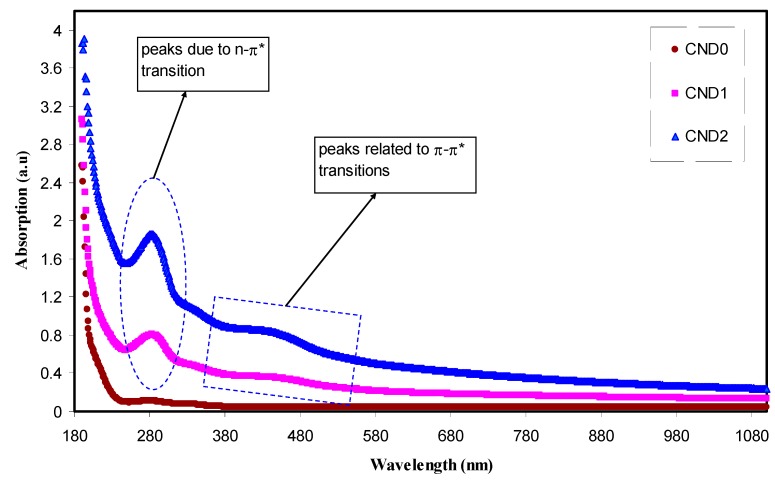
Absorption spectra for all the films. Clearly with increasing CNDs concentration the absorption shifts to higher wavelengths.

**Figure 6:**
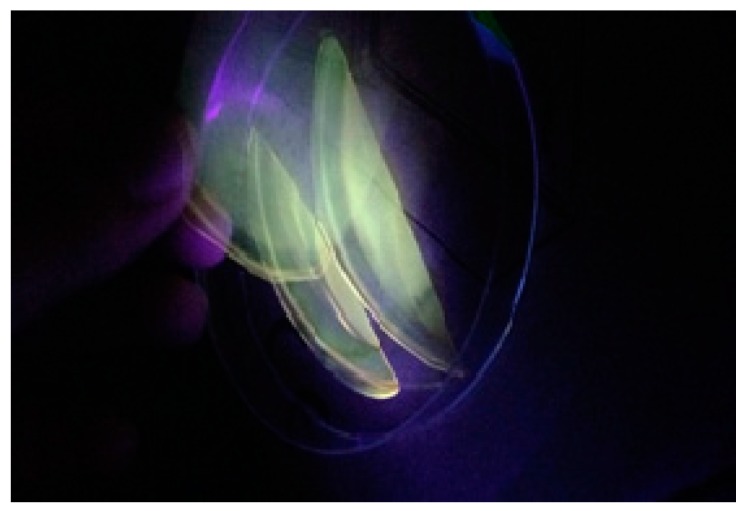
The digital photograph of CND2 sample, under UV light exposure.

Figure 5 shows the absorption spectra of both pure PVA and PVA:CND film. It is clearly seen that two distinguish peaks have manifested in the UV region. Compared to pure PVA, there is a tuned UV absorption region in the doped ones. The absorption of light in the UV and visible regions result in electron promotion in σ and π and n-orbitals from the ground state to the higher excited state as described by molecular orbital theory. As a consequence, σ → σ*, n → π*, and π → π* occur. Most optical transitions are taking place in the visible region caused by impurities. As a result, the generation of defects are color centered [34]. Figure 5 shows detection of two peaks at 280 nm and 430 nm owing to the n-π* and π-π* transitions, respectively [35,36,37]. Applications in various fields, such as biosensors, imaging probes, viral capsids, QD-based laser, light emitting devices (LEDs) and photovoltaic cells can be ascribed to the unique optical and electronic properties of CDs particles [38]. It is obvious that the onset absorption of the PVA:CND composites is from 580 nm, which lies in the visible region. The lower-mid region of the visible spectrum of the CNDs with their tunable absorption is vital for applications in optoelectronics and sensors to new formulation of bioimaging assays [39]. It is extremely interesting to notice that as the concentration of CNDs increases, the absorption intensities of n-π* and π-π* transitions are also increased. This can strongly be related to the high density of both –OH and –NH_2_ groups on the CNDs surface [4,9,37]. To check the fluorescence behavior of the PVA:CNDs composite samples, a UV lamp was used in a dark box. Figure 6 exhibits a digital photograph of apparent yellow luminescence of the composite under UV exposure.

**Figure 7:**
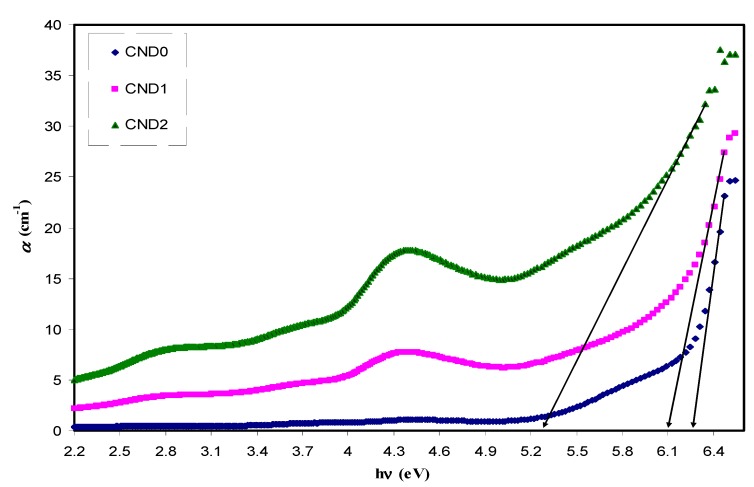
Absorption coefficient versus photon energy for all the films. Clearly with increasing CNDs concentration the absorption edge shifts lower photon energy.

UV-vis is an informative technique for studying the electronic transitions. Band strength or band-gap energy can be measured from absorption edge in crystalline and non-crystalline materials [40]. The absorption edge is a region in which an electron is jumped from a lower energy state to a higher energy state by an incident photon. The following equation was used to calculate the optical absorption coefficient from the transmittance and reflectance spectra of the films [41]:

(2) $$\alpha =\frac{1}{t}Ln\left(\frac{T}{{\left(1-R\right)}^{2}}\right)$$

where *t*, *T* and *R* are the thickness, transmittance and reflectance of the sample, respectively. Gradually increasing the absorption coefficient with applied photon energies indicates the amorphous nature of the samples [41]. Figure 7 presents the absorption coefficient as a function of photon energy for pure PVA and PVA:CNDs films. A clear red shift from 6.2 eV to 5.3 eV corresponds to the absorption edge. Clearly, with an increase in CNDs concentration the absorption edge shifts towards lower photon energy. The shift in absorption edge might have resulted from the formation of conjugated bond system caused by bond cleavage and reconstruction. This supports the structural and chemical modifications of PVA incorporated with CNDs particles [42].

**Figure 8:**
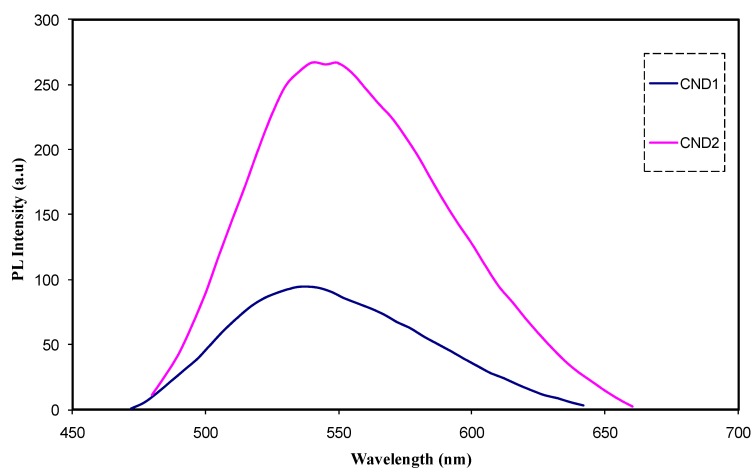
PL spectra for PVA:CNDs composite films at excitation wavelength of 431 nm.

Figure 8 shows the PL spectra for the PVA:CND composite samples at excitation wavelength of 431 nm. Recently, a great deal of research has been devoted to PL of C-dots which is one of characteristics of C-dots that applied in the photocatalysis. The Stokes type emission is obeyed by PL emission, which has a longer wavelength than the excitation one [2]. This emission formed after absorption of photons (electromagnetic radiation). The relaxing and cooling of carrier distribution led to a decrease in width of the PL peak and also to emission energy shifts to match the ground state of the exaction. As the carrier density is increased, the appearance of additional peaks from higher sub-band transitions occur and an increase in the excitation density changes the whole emission spectra [43]. It is obviously noted that as the concentration of C-dot increased, the PL peak intensity increases. This UV absorption resulted in the appearance of characteristic peaks in PL spectra. It is well-known that the excitation UV has shorter wavelength than the emission. It is documented that emission of 385 nm is resulted from the extended conjugation of π-electron domains (island) present in C-dots. The presence of surface trap states (STS) in C-dots led to a strong emission at 460 nm. Recently, C-dots have been characteristic with the existence of STS that contributes in electronic conduction. Clearly, accommodation of electrons in STS facilitates emission at 460 nm; as a consequence, it determines the electronic nature of C-dots [1]. It is well-defined that most PL emission observations can be classified to some extent into two main categories; firstly, one is owing to band gap transitions resulted from conjugated π-domains and secondly, it is due to the defects in the graphene structures. The two factors are synergic in many cases. More clearly, the exploitation and manipulation of defects in graphene sheets results in the creation or induction of the p-domains [2].

### 4.4. Refractive Index and Optical Dielectric Constant Studies

Today, studies on the electrical and optical properties of polymers have shown a great deal in view of their applications in optical devices with remarkable reflection, antireflection, interference and polarization properties. The optical properties of polymers can be properly modified by the addition of dopants depending on their reactivity with the host matrix [26,44,45]. One of the parameters is the optical refractive index (*n*), which is the measure of the reduction rate of the speed of light in the medium. The refractive index of the samples has been calculated from the reflectance (*R*) and extinction coefficient (*K*) by using the following equation [26],

(3) $$n=\left[\frac{\left(1+R\right)}{\left(1-R\right)}\right]+\sqrt{\frac{4\times R}{{\left(1-R\right)}^{2}}}-K^{2}$$

From the following mathematical relationship:

*K = αλ/4πt*

The extinction coefficient, *K* is directly proportional to both absorption coefficient (*α*) and *λ* is the wavelength whereas inversely proportional to the sample thickness (*t*).

In another mathematical expression, one can clearly see that the reflectance (*R*) can be computed from the absorption (*A*) and transmittance (*T*) values (*R = 1−(A + T*)). The *T* values are calculated from Beer’s law (*T = 10^−A^*). 

**Figure 9:**
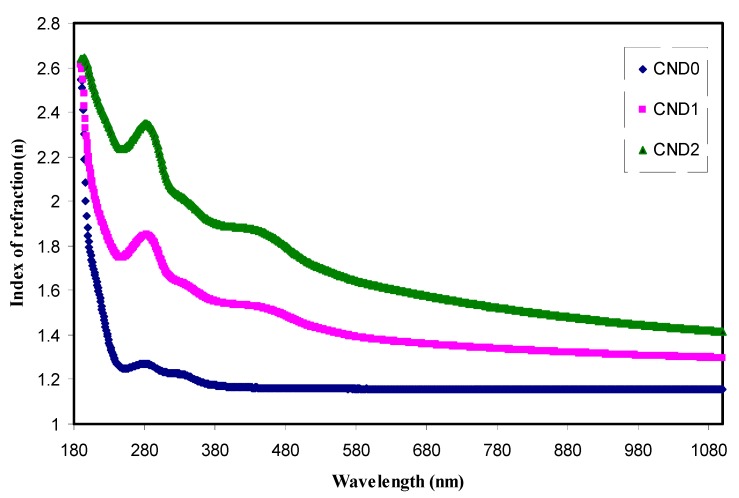
The index of refraction versus wavelength for all the films. Clearly with increasing CNDs concentration the dispersion increased.

From Figure 9, the refractive index spectra of pure PVA and the doped samples are shown. It is apparently revealed that there is a direct proportionality between refractive index and CND concentration. The value of the refractive index is greater than one because of slowing down of photons as a result of interaction with electrons of the host material. It is well-known that the phase velocity of light in vacuum is c = 2.99 × 10^8^ m/s equals the group velocity which is independent of the optical frequency. In contrast, it is typically smaller by a factor n, called the refractive index, in a medium which is frequency dependent. Thereby, the higher value of refractive index of PVA:CND composite the more slowing down occurs of the phase velocity. This is due to the fact that film incorporated with C-dots results in an increase in density and as a consequence, the refractive index becoming increasing predictable [46]. This is also relevant to fact that the refractive index is a function of density, which in other words, is related to the polarizability of the medium [47]. Moreover, for the all doped films, the dispersion of the refractive index versus wavelength is observed compared to the pure PVA. This dispersion behavior of *n* in the doped samples can be related to the increase of density. For additional interpretation, two typical peaks are seen in the spectra of refractive index of the composite samples which can be correlated to aromatic π-π* and n-π* transitions, corresponding to C=C, C=O respectively [48]. It is worth noticing that as the CND concentration increases, the peak intensity increases as a result of populations of more electrons and the number of surface groups. In an attempt to estimate the refractive index of the films, the long wavelength region was extended to Y-axis. 

**Figure 10:**
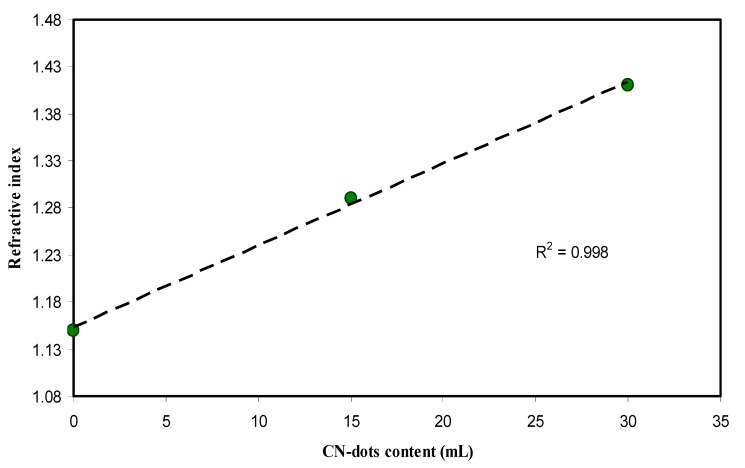
The index of refraction versus CNDs concentration. The linear increase reveals the homogeneous dispersion of CNDs particles.

**Figure 11:**
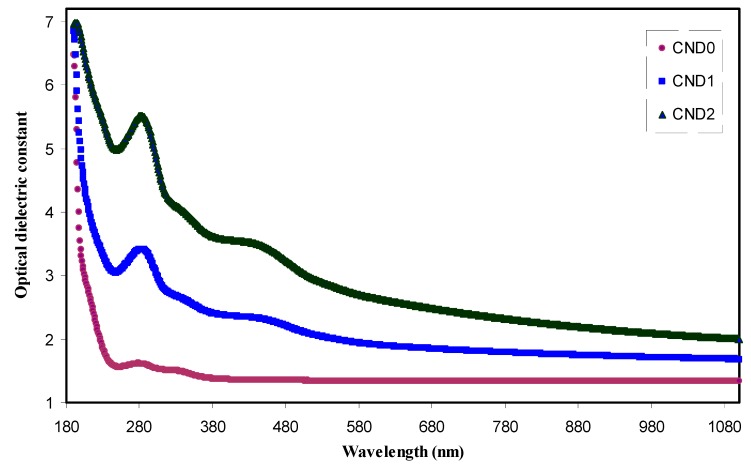
The optical dielectric constant versus wavelength for all the films. Clearly with increasing CNDs concentration the ɛ’ increased.

Figure 10 shows the plot between refractive index and CND concentrations. This linearity in the plot is reported in several studies and considered as a satisfactory dispersion of fillers throughout the polymer matrix [18,49,50,51,52]. In the data analysis, the r^2^ value was determined to be 0.99 from the fitted regression line. This indicated the homogeneity of dispersion of CND throughout the PVA polymer. Furthermore, it is proved to large extend that the refractive index is related to optical dielectric constant (*ɛ*_1_) parameter which directly related to the localization of electronic states within the forbidden gap of materials [18,22,53]:

(4) $$\varepsilon_{1}=n^{2}-K^{2}=\varepsilon_{\infty }-\frac{e^{2}}{4\pi C^{2}\varepsilon_{o}}\frac{N}{m^{*}}\lambda^{2}$$

where *ɛ_∞_* and *ɛ_o_* are the dielectric constant at higher wavelengths and the free space dielectric constant, respectively. N/m* is the ratio of localized electronic state density to the effective mass, *K* is the extinction coefficient and *e* and *C* have their usual meanings. Figure 11 exhibits the variation of the optical dielectric constant (*ɛ*_1_) with wavelength at different CNDs concentrations. The *ɛ*_1_ value and CND concentration have direct proportionality. An increase in *ɛ*_1_ value from 1.3 to 2.4 can be ascribed to the increment of the density of states because of direct correlation of *ɛ*_1_ parameter to the density of states inside the forbidden gap of the solid polymer films [22]. The relationship between the static dielectric constant (*ɛ*_0_) within long wavelengths is well reported [54]. Accordingly, Penn model explains the optical dielectric constant that can strongly be correlated with optical band gap (*E_0_*) [55] as follows:

(5) $$\varepsilon_{\left(o\right)}\approx 1+{\left(\hbar \omega_{p}/E_{o}\right)}^{2}$$

However, this model has involved the refractive index (*n*) considering ɛ = *n^2^*. Thus, the Penn model should be expressed on the basis of the refractive index [56].

### 4.5. Bandgap Study

An Interband absorption process deals with transition of electrons between the bands of solid materials. The absorption edge originated from the onset of optical transitions across the fundamental band gap [57]. Our recent achievements revealed that the fundamental absorption edge derived from the dielectric loss represents the energy bandgap [18,41,44,51,52,58]. These are supported by quantum methods for bandgap investigations. Knowledge of both real and imaginary parts of the dielectric function allows calculating important optical functions [59]. The following complex optical dielectric function is usually used to describe the optical properties of a solid material, interrelated with photon and electron interactions [60].

ɛ* = *ɛ*_1(*ω*)_ + *jɛ*_2(*ω*)_ (6)

**Figure 12:**
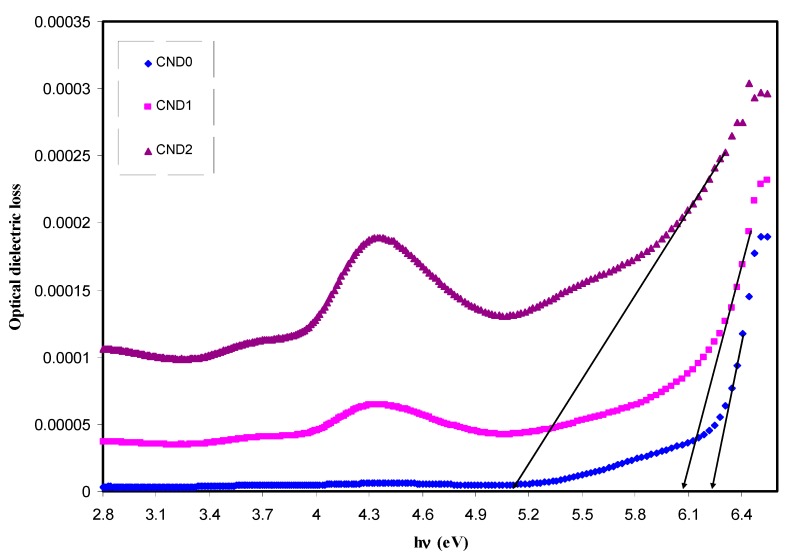
Optical dielectric loss versus photon energy (hυ) for all samples. Distinguishable linear parts can be manifested at high photon energy region which can be used to estimate the optical band gap.

*ε*_1(*ω*)_ as real part and *ε*_2(*ω*)_ as imaginary part of the complex are associated with electronic polarizability and electronic absorption of the material, respectively [61]. To better understand the electronic structure of different materials, the optical functions have to be studied. Frequency-dependent dielectric function is closely linked to the electronic band structure. Accordingly, the optical properties of homogeneous mediums at all photon energies can be characterized [62]. From the quantum mechanical aspect, the transitions between occupied and unoccupied states are highly related to optical dielectric loss parameter [18,41,44,51,52,58,62]. The equation below can directly be used to determine the imaginary part *ε*_2(*ω*)_ of the complex dielectric function from the momentum matrix elements between the occupied and the unoccupied electronic states.

(7) $$\varepsilon_{2}=\frac{2\pi e^{2}}{\Omega \varepsilon_{o}}\sum_{v,c,k}{\left|\langle \Psi_{k}^{c}\left|\vec{u}\cdot \vec{r}\right|\Psi_{k}^{v}\rangle \right|}^{2}\delta \left(E_{k}^{c}-E_{k}^{v}-\hbar \omega \right)$$

where ω is the frequency of light, e the electronic charge, $\vec{u}$ the vector defining the polarization of the incident electric field, and ($E_{k}^{c}$)and ($E_{k}^{v}$)the conduction and valence band wave functions at k, respectively [62]. It is well documented that the fundamental absorption edge in optical dielectric loss spectra provides the optical band gap [63]. A rapid rise near the absorption edge can be a direct evidence for band gap determination [64,65]. The main contributions to the optical spectra are derived from the top valence band (VB) to the lower conduction bands (CB), known as the fundamental absorption edge. The critical points obtained are associated with the band-gap values [60]. Metallic, semiconducting or insulating characteristics of a material can be determined from the electronic properties [66]. Figure 12 reveals the plot between the optical dielectric loss and photon energy for all the samples. The intersection of linear part of ɛ_2_ with the photon energy axis was exploited to estimate the optical band gaps. The estimated values from the plot are listed in Table 1. At a first glance, the higher CNDs concentration, the band gap is reduced. From previous studies, it was concluded that the polymer composites with reduced optical band gap are crucial for photovoltaic and other optoelectronic applications. Huang et. al., reported that the power conversion efficiency of almost 12% of polymer-fullerene-based bulk hetero-junction solar cell incorporated with C-dot particles is increased as a consequence of effective light conversion of near ultraviolet and blue-violet portions of sunlight [67]. Earlier study documented that C-Dot/polymer composites showed relatively acceptable photo-stability, and thus can be used as environmentally friendly composite, low-cost phosphors for solid-state lighting and wave guide applications [68]. Another important aspect in band gap study is the specification of the type of electronic transition. When a photon with a sufficient energy absorbed by an electron transition occurs from the top of the valence band to the bottom of the conduction band and the electron transition obeys some quantum mechanical rules. Tauc’s method was applied to specify the type of electronic transition. Optical absorption spectrum is significant for studying the physical properties of polymers comprising the study of a band construction and electronic properties when at pure and doped states [69]. In fact, the optical band gap was estimated from the data of absorption coefficient versus wavelength using Tauc’s equation.

**Figure 13:**
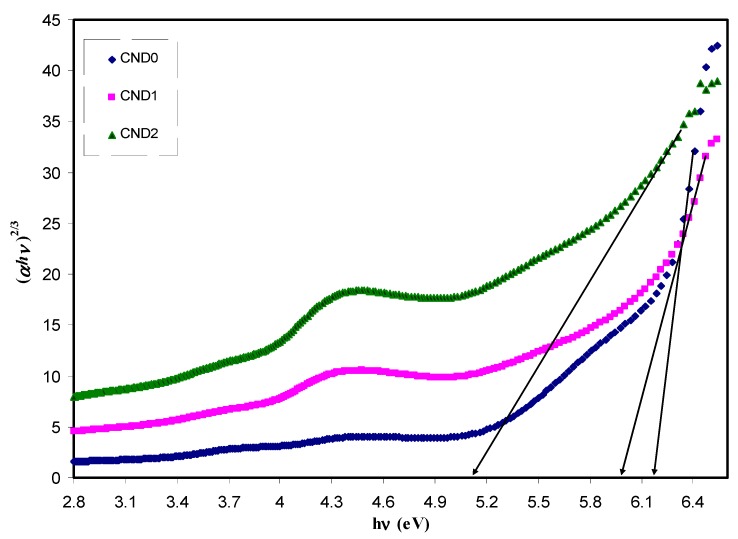
Plot of (*αhυ*)^2/3^ versus photon energy (*hυ*) for all the samples.

**Figure 14:**
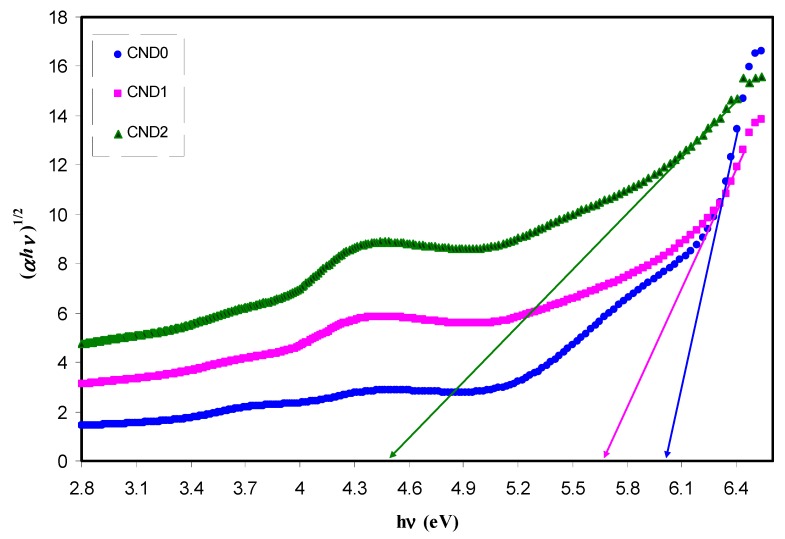
Plot of (*αhυ*)^1/3^ versus photon energy (*hυ*) for all the samples.

**Figure 15:**
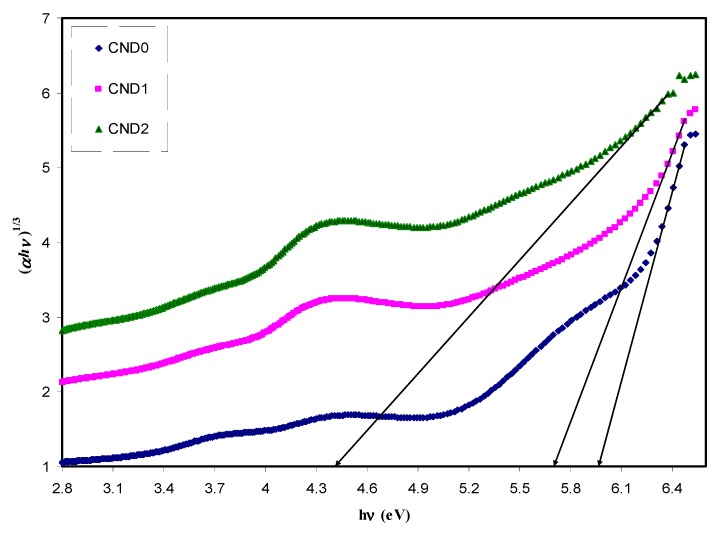
The plots of (*αhυ*)^^1/3^ vs (*hυ*) for all the samples.

*αhv = B(hv − E_g_)^n^* (8)

where *α*, *hυ*, *B* and *E_g_* denote the absorption coefficient, the photon energy, the band form parameter and the optical bandgap of the samples, respectively, and *n* a constant, being related to the density of states distribution and determined the type of transition. The *n* values are equal to 1/2 and 3/2 for direct allowed and forbidden transitions, respectively, while *n* values of 2 and 3 are for the case of indirect allowed and forbidden transitions, respectively [58]. A direct transition proceeds when the electrons wave vector remains unchanged. However, the interaction with a lattice vibration occurs in the indirect transition, in which the lowest region of the CB locates at different part of the k-space from the highest region of the VB [69]. The plot of (αhv)^1/n^ versus photon energy (hv) when n = 3/2, 2 and 3 is shown in Figure 13, Figure 14 and Figure 15. In the data analysis, from these figures, various optical band gaps were estimated and tabulated in Table 1. The results showed that the band gaps of the composite films are reduced compared to pure PVA, as a consequence of insulating properties of PVA. Variations in the band gap values make hard to identify a dominant type of electronic transition. In order to specify the exact type of electronic transition in the samples, the band gaps obtained from Tauc’s method (i.e., Figure 13, Figure 14 and Figure 15) were compared to the optical band gaps derived from optical dielectric loss plot (i.e., Figure 12). As a result of comparison, the type of forbidden direct transition (i.e., n = 3/2) can be deduced. To determine the band gap and understand the electron transition phenomena from the top of VB to the bottom of CB both optical dielectric loss parameter and Tauc’s model have to be tested. In our previous works, the use of optical dielectric loss parameter for studying the bandgap has been well established [18,41,44,51,52,58,69]. Thus, the present work is an additional support to our hypothesis implying that optical dielectric loss parameter and Tauc’s model are sufficient to study the band gap and electron transition types, respectively. From Table 1 it is clear that the band gap decreased upon the addition of CNDs to PVA. A decrease in *E_g_* upon increasing CND concentration might result from the creation of a higher number of free charge carriers/radicals [42]. From the results, one can conclude that these samples can be used for various optoelectronics and previous studies revealed the use of carbon particles in various applications. Huang et. al. [70], used the hybrid materials of carbon and graphene for the fabrication flexible strain sensors and Ke et. al. [71], used carbon dots as fluorescent sensors. Moreover, the capacitance value of supercapacitors is increased upon incorporation of carbon dots to the electrode materials [72]. Thus, CNDs particles have a wide application from optoelectronics to electrochemical devices.

## 5. Conclusions

In conclusions, fabrication of PVA:CNDs is fascinating after characterizations using a range of spectroscopic techniques. Changes in the FTIR spectral features indicated a good cross-linking between PVA and CND nano-particles. Shifts in XRD spectra of the composites confirmed the complex formation between them. The Debye-Scherrer formula was used to calculate the crystallite size of CNDs and crystalline area of pure PVA and PVA:CNDs composites. The crystallite size of PVA was reduced upon the addition of CNDs particles. Furthermore, the effect of these nano-particles on tuning the absorption of UV spectra in the nano-composites was studied. Strong absorptions at 280 and 330 nm were assigned to n-π* and π-π* transition. A reduction in the optical band gap resulted from a shift in absorption edge to lower photon energy. The existence and dispersion of C-dots with different sizes on the surface of composites films were proved using FESEM images. The luminescence behavior of PVA:CND composites was verified using digital photograph and photo luminescence (PL) measurement and the PL intensity increased with an increase in CNDs concentrations. A linear increase of the refractive index with increasing CND concentration revealed a homogeneous distribution of the particles throughout the host PVA. Optical dielectric loss parameter was utilized to estimate the optical band gap. The study of Tauc’s model established a forbidden direct type of electronic transition. The results of the present work are an additional support to our hypothesis implying that optical dielectric loss parameter and Tauc’s model are sufficient to study the bandgap and electron transition types, respectively.

## Figures and Tables

**Table 1:** Estimated bandgap from Taucs model and optical dielectric loss plots.

Sample Designation	*E_g_*(eV) from Tauc Method (*n* = 3)	*E_g_*(eV) from Tauc Method (*n* = 2)	*E_g_* (eV) from Tauc Method (*n* = 3/2)	Estimated Bandgap from ɛ″-hυ Plot
CND0	5.95	6	6.18	6.2
CND1	5.7	5.67	5.96	6.05
CND2	4.4	4.5	5.12	5.12
